# Brain Arteriovenous Malformation Hemorrhage and Pituitary Adenoma in a COVID-19-Positive Patient

**DOI:** 10.7759/cureus.67644

**Published:** 2024-08-23

**Authors:** Edgar Nathal, Eliezer Villanueva-Castro, Alma Ortiz-Plata, Alejandro Serrano-Rubio, Martha Lilia Tena Suck

**Affiliations:** 1 Department of Neurosurgery, Instituto Nacional de Neurología y Neurocirugía Manuel Velasco Suárez, Mexico City, MEX; 2 Laboratory of Experimental Neuropathology, Instituto Nacional de Neurología y Neurocirugía Manuel Velasco Suárez, Mexico City, MEX; 3 Department of Neuropathology, Instituto Nacional de Neurología y Neurocirugía Manuel Velasco Suárez, Mexico City, MEX

**Keywords:** covid-19, cerebrovascular disease, pituitary apoplexy, pituitary adenoma, brain arteriovenous malformation

## Abstract

Brain arteriovenous malformations (AVMs) are usually asymptomatic. They can cause intense pain or bleeding or lead to other serious medical problems. We present a rare case of a woman who presented with a severe headache and was brought to the emergency service for an intracerebral hemorrhage due to a ruptured AVM. During the surgery, a sellar mass was identified that was also resected. AVM showed vasculitis, endarteritis, endothelial damage, leukocyte plug, and damage to the vessel wall with fragmentation of the collagen and actin filaments. The sellar mass showed a non-functioning pituitary adenoma with hemorrhagic foci and necrosis as well as a proteinaceous vs. lipid material deposition with minimal vascular changes such as endothelial hyperplasia with minimal vasculitis and hyperplasia of reticular stellate cells, with positive glial fibrillary acidic protein (GFAP), which expressed low expression of luteinizing hormone (LH) and follicle-stimulating hormone (FSH), IL6, IL10, IL17, tumor necrosis factor-alpha (TNFa), HIF1a, factor VIII (FVIII), platelet-derived growth factor (PDGF), vascular endothelial growth factor (VEGF), and VEGF receptor 2 (VEGFR2). The patient's polymerase chain reaction COVID-19 test was positive, and she died three days after the surgery procedure. In our knowledge of COVID-19 brain lesions and in the literature review, this was a rare case of a double pathology associated with COVID-19 infection characterized by rupture of the AVM with hemorrhages and brain infarcts associated with endarteritis, vessel wall injuries, and pituitary apoplexy.

## Introduction

COVID-19 has been linked to cerebrovascular incidents, including intracerebral hemorrhages, in individuals with and without prior neurological conditions. The virus-triggered cytokine storm and resulting endothelial dysfunction are believed to enhance vascular permeability, thereby increasing the risk of hemorrhages, particularly in brain regions already compromised by tumors. The SARS-CoV-2 infection can damage the central nervous system through mechanisms such as hypoxia, widespread inflammation, and the activation of the renin-angiotensin-aldosterone system. These processes may worsen pre-existing conditions, like brain tumors, making intracerebral hemorrhages more likely [1,2].

Brain arteriovenous malformations (AVMs) are anomalies of the cerebral vascular system, consisting of tortuous veins and arteries without a capillary bed in between. The most prevalent kind of intracranial vascular malformation and the main factor contributing to nontraumatic intracerebral hemorrhages in young individuals under the age of 35 is an AVM [3]. AVMs have an annual incidence of roughly one per 100,000 in an unselected population [4], and they are the causative lesion for 4% of all primary intracerebral hemorrhages, up to one-third in young adults, 1% to 2% of all strokes, and 3% of strokes in young adults [5].

The vascular nidus of an AVM is characterized by shunting through a collection of tortuous dysmorphic vessels, with blood flowing directly from arteries into veins. Bleeding can occur from the draining veins or from arterial aneurysms arising as a result of this increased pressure [6]. There are many classifications, and they are often graded according to size, venous drainage (deep or superficial), and the eloquence of surrounding tissue (e.g., Spetzler-Martin grading scale). The clinical course can be either asymptomatic or associated with headaches, seizures, or sudden neurological impairment, dependent on the location of the bleeding [3]. The risk of spontaneous bleeding in unruptured lesions is as low as 1% per year [7], and the morbidity can be as high as 53% to 81% [7], with mortality ranging from 10% to 30% [8].

The pathological features of a ruptured brain AVM include the presence of abnormally enlarged and twisted blood vessels, which are susceptible to rupture due to the absence of a normal capillary interface between arteries and veins. Upon examination, these ruptured AVMs typically show signs of necrosis and hemorrhage within the affected area, along with indications of previous bleeding episodes, such as macrophages containing hemosiderin. The vessel walls within the AVM may exhibit various degrees of fibrosis, hyalinization, and sometimes inflammatory cell infiltration, all of which contribute to vessel fragility and an increased risk of rupture [1,2].

Pituitary apoplexy is a medical emergency characterized by the sudden onset of hemorrhage or infarction within the pituitary gland, usually in the context of a pre-existing pituitary adenoma. It leads to a rapid expansion of the tumor, resulting in increased intracranial pressure and acute symptoms such as severe headache, visual disturbances (e.g., bitemporal hemianopia), ophthalmoplegia, and altered mental status. This condition may also cause acute adrenal insufficiency, which can be life-threatening if not promptly addressed [9,10].

The pathological aspect of a ruptured brain AVM is also well known, but our case report illustrates an acute rupture of a lesion in a patient infected with SARS-CoV-2 (COVID-19), in association with pituitary apoplexy.

## Case presentation

This 46-year-old woman had a previously unremarkable medical history except for high blood pressure, which was managed with an angiotensin II receptor blocker (ARB). The patient presented with an acute headache, visual disturbances, and right hemiplegia four hours prior to arriving at the emergency room, along with an inability to produce speech. On examination, the patient exhibited spontaneous eye opening, a nonverbal response due to motor aphasia, and was able to follow simple commands with a Glasgow Coma Scale score of 11 (eye 4, verbal 1, and motor 6). The patient could move the right side of the body in response to verbal commands with a strength of 2/5 on the Daniels muscle grading scale, while the left side exhibited full strength at 5/5. Visual field examination revealed new-onset bitemporal hemianopia; however, the evaluation was challenging due to the presence of motor aphasia.

**Figure 1:**
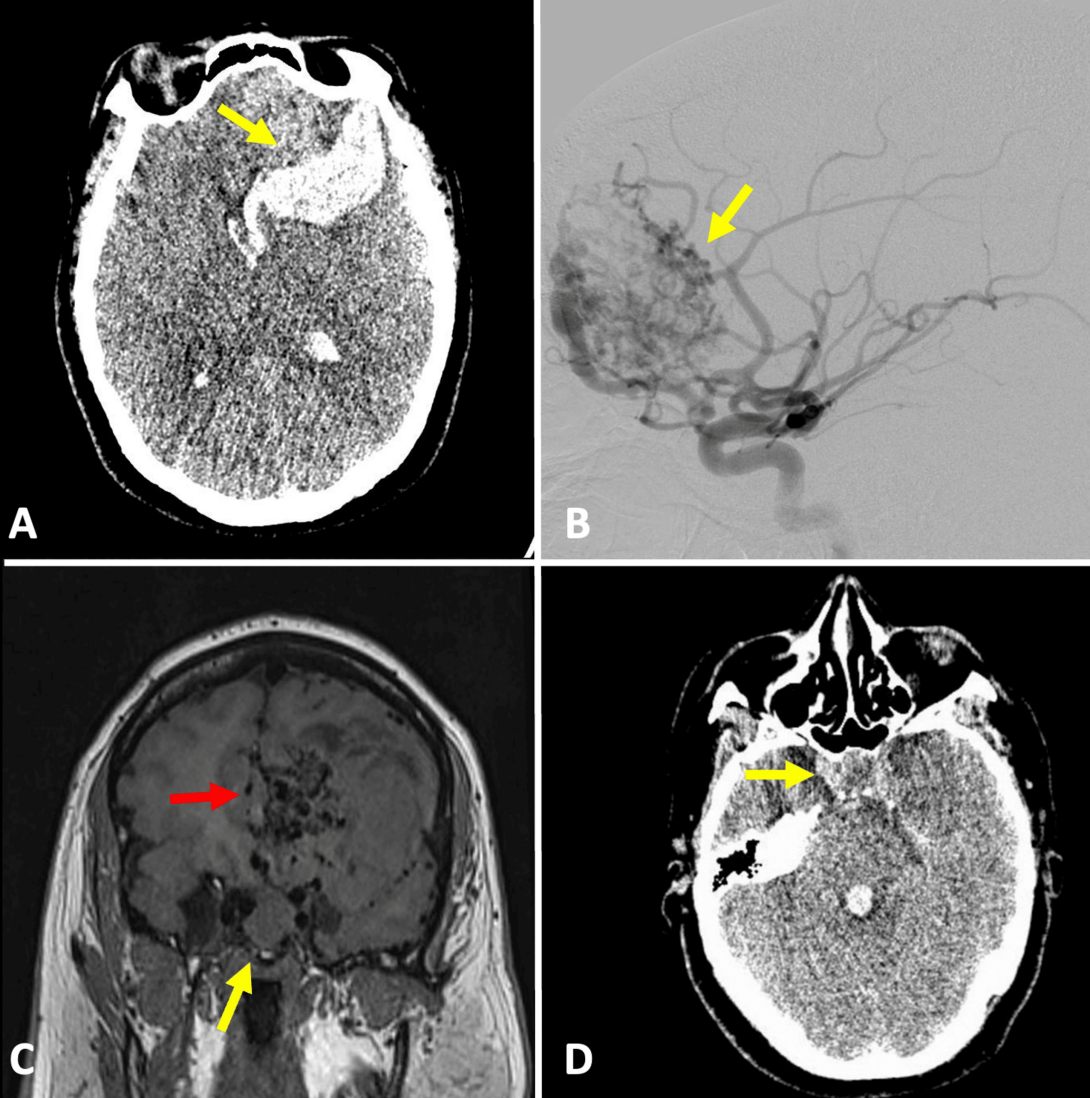
Preoperative imaging studies. (A) An axial non-contrast CT image of the head shows an intracerebral hemorrhage (yellow arrow). (B) Cerebral angiography reveals an arteriovenous malformation, Spetzler-Martin grade III (yellow arrow). (C) A coronal MRI image demonstrates the presence of a pituitary macroadenoma (yellow arrow) and a left frontal arteriovenous malformation (red arrow). (D) An axial non-contrast CT image of the head shows a heterogeneous component in the sellar region (yellow arrow).

**Figure 2:**
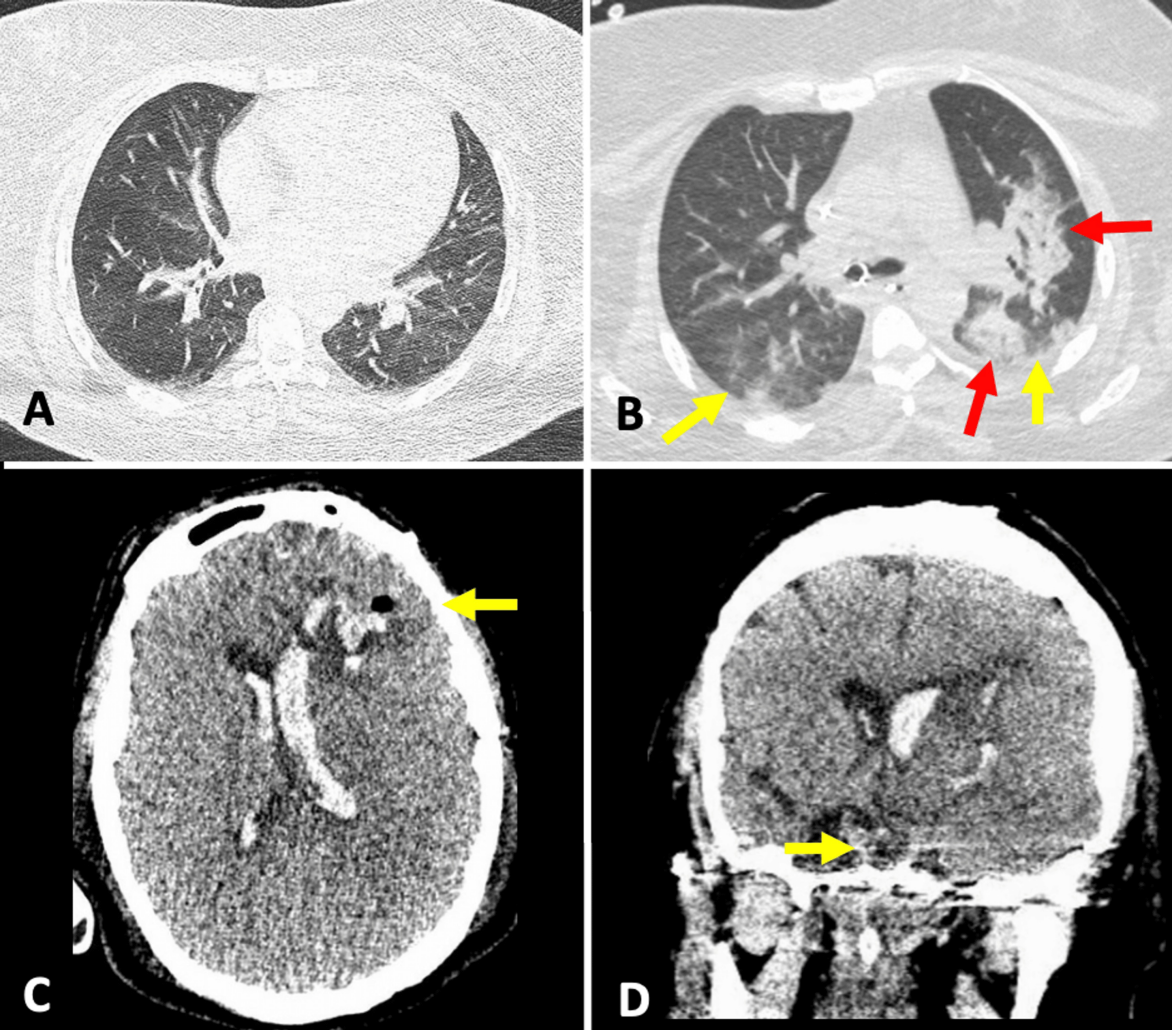
Computed tomography images. (A) Axial computed tomography (CT) image of the lung taken preoperatively. (B) Axial CT image of the lung taken postoperatively during respiratory deterioration in the patient, showing bilateral ground-glass opacity with peripheral predominance (yellow arrows) and foci of consolidation (red arrows). (C) Axial and coronal (D) CT images of the postoperative head show expected surgical changes (yellow arrows).

The imaging workup revealed a subarachnoid hemorrhage accompanied by a left frontal intraparenchymal hemorrhage that had ruptured into the ventricular system. Preoperative MRI and catheter-based angiography identified an ipsilateral frontal AVM (Spetzler-Martin grade III) and a pituitary macroadenoma (PA) (Figure 1). An emergency surgical evacuation of the parenchymal hematoma, followed by the removal of the AVM, was performed transcortically through the middle frontal gyrus. After the complete drainage of the hematoma and during the AVM resection, feeders originating from branches of the left anterior cerebral artery were coagulated, with the lesion exhibiting only superficial drainage to the superior sagittal sinus. The AVM was successfully resected without complications. At the conclusion of the hematoma drainage and AVM resection, which were the causes of the patient’s acute neurological condition, the patient remained stable under neuroanesthesiology monitoring throughout the surgical procedure. Given the presence of a sellar tumor without a histopathological diagnosis and the occurrence of intraventricular bleeding, the neurosurgeon opted for an interoptic approach. This involved draining the interoptic cistern and fenestrating the lamina terminalis to facilitate communication between the ventricular system and the subarachnoid space, followed by intracapsular resection of the sellar tumor. The early postoperative course was uneventful, and within a few hours, the diagnosis of COVID-19 infection was confirmed via the preoperative nasopharyngeal swab. The patient had no prior symptoms of COVID-19, and the sample was taken as part of a screening protocol due to a new outbreak of COVID-19. Laboratory tests showed normal values in the first hours after surgery, including arterial blood gas analysis and a hormonal profile with thyroid-stimulating hormone at 0.538 mIU/L, free T4 at 14.8 pmol/L, cortisol at 30.4 mcg/dL, and prolactin at 6.5 ng/mL. The patient’s respiratory status gradually deteriorated (Figure 2). We did not have access to antiviral drugs for the management of complicated COVID-19; however, the patient was treated with steroids (dexamethasone) and prophylactic doses of anticoagulants (enoxaparin). Although bronchial secretion cultures were negative, antibiotic coverage with ceftriaxone and clindamycin was maintained due to the presence of pulmonary consolidations. Despite these medical interventions, the patient required increasing levels of oxygen support through invasive ventilation and showed worsening hypoxemia, as indicated by blood gas analysis. A cranial CT scan was performed to rule out hydrocephalus (Figure 2), and the pupillary diameter was continuously monitored. Unfortunately, the patient passed away four days later due to pulmonary complications from COVID-19 pneumonia. The family declined an autopsy.

**Figure 3:**
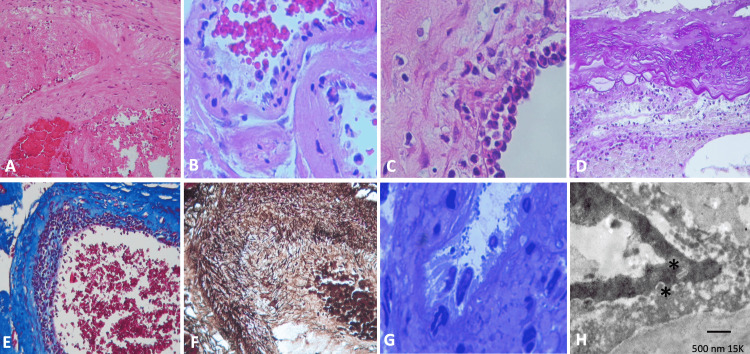
Histological features and electron microscopy of the arteriovenous malformation. (A) Abnormal vascular dilatation was observed. Some vessels displayed a single layer of endothelium with a thin collagenous wall. Necrosis and bleeding were noted, as well as malformed capillaries, arteries, and venules with abrupt changes in the thickness of the medial and elastic layers of vessels (hematoxylin & eosin (H&E), x5). (B) Some hyalinized thin-walled vessels have atypical endothelial cells and necrosis (H&E, x20). (C) Loss of endothelial cells with the presence of inflammatory cells (H&E, x40) is observed in some vessels. (D) Periodic acid-Schiff (PAS) staining and fragmentation of large vessels (PAS, x20) are better observed. (E) Masson stain was observed in blue vessels with necrosis, fibrin, and inflammation (H&E, x20). (F) Reticulin stain showed fragmentation of the fibers and loss of them (x5). (G) A semithin section stained with toluidine blue stain showed hyperplastic endothelial cells, nuclei with apoptotic morphology, and retrograde migration changes (x100). Transmission electron microscopy (H) revealed granular material at the luminal border (*) (500 nm, 15K).

**Figure 4:**
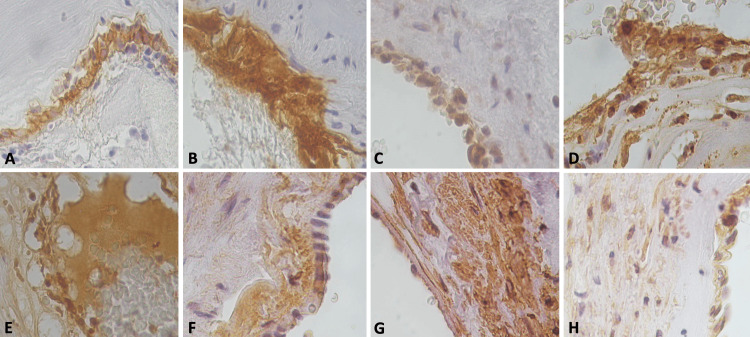
Immunohistochemistry of the arteriovenous malformation. (A) CD34-positive immunoreaction in endothelial cells (x40). (B) CD31 showed overexpression in endothelial cells (x40). (C) Platelet-derived growth factor (PDGF)-positive immunoreaction in endothelial cells (x40). (D) Interleukin 6 was also positive in endothelial cells and endothelial cell migration (x40). (E) Galectin 3 was positive both in endothelial cells, inflammatory cells, and the extravasated fluid in the lumen of the vessels (x40). (F) Collagen IV shows a thick, fragmented subendothelial layer (x40). (G) Smooth muscle actin fragmentation is observed (x40), and muscle fibers (H) were also positive for PDGF (x40).

Two different specimens were processed, corresponding to each lesion. The first was a vascular lesion and an irregularly oval fragment measuring 32 x 30 x 20 mm. It was dark reddish brown in color, with a finely granular surface. The section identified a nutritive artery and plethoric congestive vessels (Figure 3). Histologically, it consisted of numerous blood vessels of varying calibers. Anomalies included the mixing of deformed blood vessels, including capillaries, arteries, and venules; these included aberrant vascular dilatation; some of the vessels showed a single layer of endothelium with a thin collagenous wall, necrosis, and bleeding (Figure 3). Capillaries are immediately adjacent to each other. They are typically not associated with enlarged feeding arteries or draining veins. However, it was observed that in some vessels, there was hyperplasia of endothelial cells; these were large with hyperchromatic nuclei (Figure 3B). Inflammatory cells of the polymorphonuclear leukocyte type were destroying and displacing the endothelial cells (Figure 3C). The periodic acid-Schiff (PAS) stain in red (Figure 3D) and Masson’s trichrome stain in blue (Figure 3E) showed loss of basal membrane with necrosis of the cells, as well as debris and inflammatory cells of the polymorphonuclear leukocyte type. The wall of the great vessels showed variable changes in edema and destruction of the vascular wall; reticulin staining showed fragmentation of the different layers of the vessels and proliferation of the vasa vasorum (Figure 3F). In the trapped brain tissue, small vessels with vascular damage and inflammatory cells were observed on the wall of the same gliotic reaction (Figures 3G, 3H). Immunohistochemical stains were performed. Endothelial cells were positive for CD34 (Figure 4A), strongly for CD31 (Figure 4B), factor VIII (FVIII), platelet-derived growth factor (PDGF) (Figure 4C), vascular endothelial growth factor (VEGF), and VEGF receptor 2 (VEGFR2), IL6 (Figure 4D), IL17, tumor necrosis factor β (TNFβ) (Figure 4E), tumor necrosis factor α (TNFα), HIF1a, galectin 1, 3, and 9 (Figure 4F) showed extravasated fluid exiting from endothelial cells. Collagen IV showed a thick, fragmented subendothelial layer (Figure 4G). With smooth muscle actin, fragmentation of muscle fibers was observed, and they were also positive for PDGF (Figure 4H), VEGF, VEGFR2, IL6, IL17, TNFkb, TNFa, and HIF1a immunopositivity reactions.

**Figure 5:**
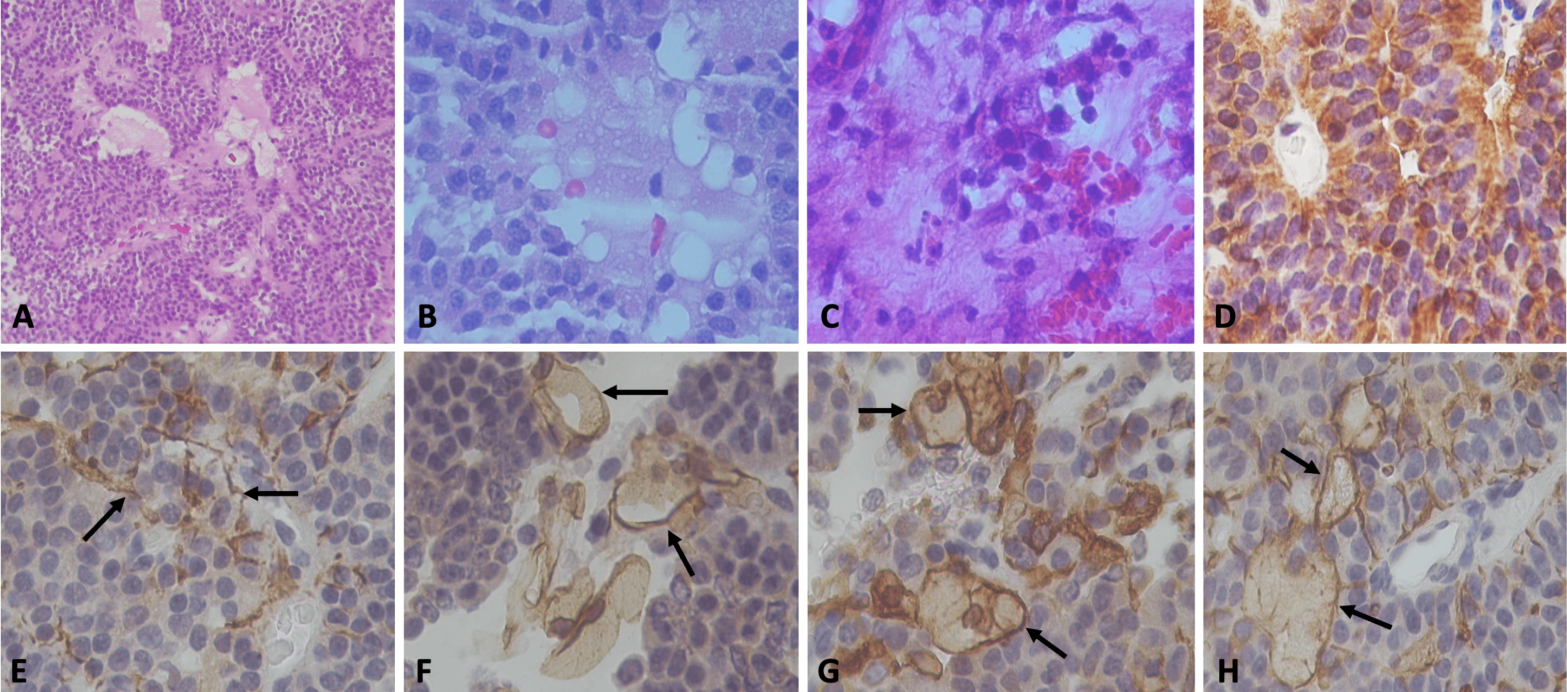
Histopathological and immunohistochemical features of pituitary adenoma. (A) A classic pituitary adenoma is identified. However, foci of hemorrhage, necrosis, and a liquid eosinophilic material were observed (hematoxylin & eosin (H&E), x10). (B) Foci of foamy material with a lipid appearance arranged diffusely with edema (H&E, x400), and in (C), we observed some blood vessels that showed endarteritis (H&E, x40). (D) By immunohistochemistry, it was positive for follicle-stimulating hormone (FSH) (x400). (E) Glial fibrillary acidic protein (GFAP) was intensely positive, showing thick fibers between epithelial cells (black arrows) (x400). (F) Vessel endothelial cells had a CD31-positive immunoreaction (black arrows) (x400). (G) Stellate cells and endothelial cells show positive expression for platelet-derived growth factor (PDGF) (black arrows) (x400) and for COVID-19 spike proteins in (H) (black arrows) (x400).

The second specimen corresponds to a sellar lesion consisting of several fragments of yellowish-white tissue with a soft, rubbery consistency. Together, they measured 10 x 7 mm. Histologically, a classic pituitary adenoma was identified. However, foci of hemorrhage and necrosis were observed (Figure 5). Foci of foamy material with a lipid appearance were arranged diffusely with edema (Figure 5B), and the blood vessels showed endarteritis (Figure 5C). By immunohistochemistry, it was positive for chromogranin, epithelial membrane antigen (EMA), cytokeratin 8, follicle-stimulating hormone (FSH), and luteinizing hormone (LH) foci (Figure 5D). Glial fibrillary acidic protein (GFAP) was intensely positive, showing thick fibers between the epithelial cells (Figure 5E), and the endothelial cells of the vessels were CD31, CD34 (Figure 5F), FVIII, PDGF (Figure 5G), VEGF, VEGFR2, IL6, IL10, HIF1a, TNFa, nuclear factor-κB (NF-κB), and IL17 with immunopositive reactions. COVID-19 spike protein showed a positive immunoreaction (Figure 5H). Prolactin, growth hormone (GH), adrenocorticotropic hormone (ACTH), thyroid-stimulating hormone (TSH), and FSH were negative. Ki67 was low (3%), and P53 was negative.

**Figure 6:**
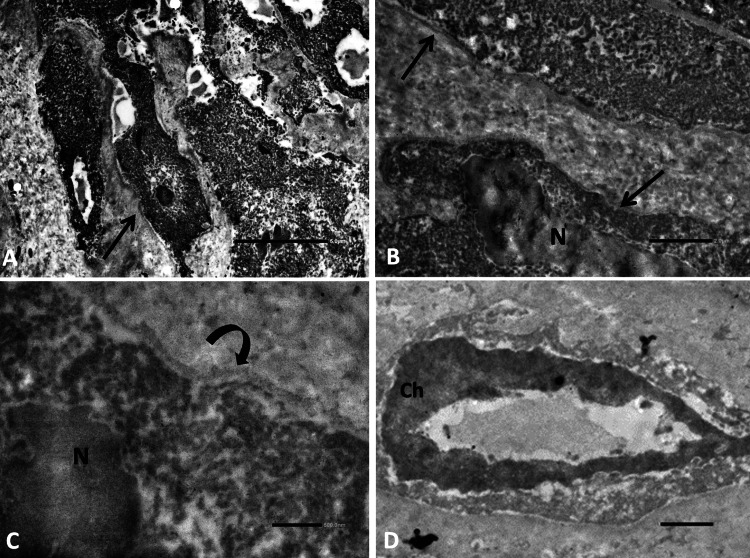
Electron microscopy of the arteriovenous malformation. (A) Endothelial cells with apparently preserved structures were found (arrow; bar scale: 5 μm; x3000). (B) Loss of intercellular junctions (arrows) were observed among endothelial cells (bar scale: 2 μm; x5000). (C) The cells showed deformed nuclei (N) and alteration of the cell membrane (curved arrow; bar scale: 500 nm; x15k). (D) Cells with signs of apoptosis were found with chromatin (Ch) condensed toward the nuclear membrane (bar scale: 1 μm; x8000). Uranyl acetate-lead citrate stain.

The ultrastructure analysis of the AVM was performed by transmission electron microscopy. Endothelial cells were observed without apparent changes (Figure 6A). However, the cells presented loss of intercellular junctions (Figure 6B), deformed nuclei, and rupture of the cell membrane (Figure 6C). Also, hyperplasic apoptosis changes in some endothelial cells were observed (Figure 6D).

COVID-19 spike proteins were detected in the adenoma (Figure 5H) but not in the AVM. Ultrastructural changes in the AVM vessels are shown in Figure 6.

## Discussion

Molecular characterization of resected AVM tissue has provided evidence for the involvement of angiogenic and inflammatory pathways. The mortality rate associated with the initial bleed is 10% [3]. The location and size of each patient’s lesion greatly affect their risk of morbidity and mortality [3,4]. Hemorrhage risk can be further exacerbated by changes in hemodynamics, such as high flow, elevated wall shear stress, and venous hypertension (VH), which can also cause inflammation, blood-brain barrier (BBB) leakage, and elevated VEGF levels [5]. Treatment with thalidomide and lenalidomide promotes the synthesis of PDGFB by endothelial cells and the recruitment of pericytes. By downregulating zonula occludens-1 and disorganizing the actin cytoskeleton, VEGF disrupts endothelium-tight junctions, increasing vascular permeability in ischemia circumstances [7]. Occludin and claudin-5 are two examples of tight junction proteins that are downregulated when VEGF levels are linked to BBB collapse [7]. Human AVMs’ aberrant arteries are subjected to varying degrees of elevated intraluminal flow and VH, which causes VEGF, HIF-1α, and Nrf2 expression to be upregulated [8]. A transcriptional factor regulates antioxidant genes and influences angiogenesis [11,12] and overexpression of PDGFA in the endothelium, media, adventitia, and perilesional tissue.

AVMs show increasing ectasia of aberrant arteries lined by flat endothelium, in contrast to the aberrant proliferation of endothelial cells in a hemangioma. Mild inflammation, subsequent dystrophic calcification, and localized thrombosis are possible [13]. PDGFB/PDGFRβ pathway crosstalk occurs in endothelial cells (ECs) [14]. PDGFB is a disulfide-linked homodimer that is secreted from the endothelium and is bound by electrostatic contacts to the extracellular matrix. Ectasia, the creation of aneurysms, and the leakage or rupture of arteries and capillaries are linked to the loss of vascular smooth muscle cells and pericytes, respectively [14]. When illustrating shunts or continuities between veins and arteries, serial sections are useful. In our case, we observed the rupture of the vessels of the malformation due to damage to the endothelial cells, hyperplasia and necrosis of the same, and the production and deposit of necrotic material and cellular debris in the endothelial cell layer and the lumen with a proliferation of inflammatory cells [13,14], with fragmentation and edema of the vessel wall, including sub-endothelial fragmentation of collagen and actin fibers.

Pathologists rely on elastic stains for making a definitive diagnosis of AVM because arteries and arterioles (with elastic lamina in their walls) are an integral part of AVM [5,6,11,12]. The collagen fibers in AVM vessels are disorganized and interrupted in the internal elastic lamina. Col-IV is a stiff fibrillar protein that provides resistance to tension, whereas Col-III forms an elastic network. Subsequent angiogenic sprouting and vessel enlargement involve PDGFB-dependent vascular smooth muscle cells/pericytes (VSMC/PC) progenitor co-migration and proliferation [13]. The blood vessel extracellular matrix is known to be broken down by matrix metalloproteinase 9 (MMP-9), which increases vascular permeability. The degree of microhemorrhage is correlated with pericyte decreases [14]. A quicker rate of blood flow through the AVM nidus is likewise correlated with a decrease in pericytes [14]. AVMs disrupt various physiological processes, such as brain angiogenesis regulation, endothelial proliferation, vascular diameter, blood flow regulation, stability of the vascular wall, and BBB integrity [14]. Vascular abnormalities including necrosis, endothelial cell injury and vasculitis, reactive fibrin and gliosis, edema, and secondary inflammation were seen in the residual brain tissue in between the vessels [11,12].

The enclosed, single-stranded, positive-sense, ribonucleic acid virus known as SARS-CoV-2 penetrates cells through the angiotensin-converting enzyme 2 (ACE2) receptors or a number of additional receptors [15]. It causes dysregulation of the ACE2 receptor, coagulopathy, uncontrolled immunological responses, and inflammation. Stroke is the most prevalent neurological illness related to COVID-19, and is disabling and tends to affect older adults and men more than women. Strokes were reported in COVID-19 individuals; of those, 68.5% were ischemic (44.5% large vessel, 24% lacunar, and 24% hemorrhagic) in the limited subset with acute neuroimaging [16].

The respiratory system is the main organ affected by COVID-19, but other organs are also impacted. There have been reports of cerebral venous thrombosis, cerebral venous sinus, deep vein thrombosis, intracranial hemorrhage, ischemic stroke, cerebral venous illness, and subarachnoid hemorrhage in the CNS [17]. Its interaction with the ACE2 receptor results in reduced perfusion in the ischemic zone and the development of bigger infarct sizes because it inactivates the receptor and disrupts blood pressure control [18]. Essentially, the pathophysiology of cerebrovascular events, such as ischemic and hemorrhagic stroke [16,18], hypoxia, and coagulopathy, reflecting microthrombi formation secondary to endothelial cell inflammation and cytokine storms, has also been linked to the impaired endothelial function in the cerebral arteries caused by ACE2 inactivation [18]. In ischemic conditions, the inflammatory response significantly exacerbates tissue injury. This process begins with the activation of leukocytes and the release of pro-inflammatory cytokines, which further intensify the initial damage by increasing vascular permeability, leading to edema and accelerating cell death. Conversely, while inflammation is also a factor in hemorrhagic events, its impact on worsening tissue damage is not as pronounced or critical as it is in ischemic conditions [19].

Arterial blockage and venous thromboembolism are caused by COVID-19-associated hypercoagulability [20]. SARS-CoV-2 infection has been linked to various neurological events, including hemorrhagic lesions in the central nervous system. Coagulopathy plays a critical role in the development of these hemorrhagic events in COVID-19 patients. The virus-induced endothelial damage and inflammatory response can lead to the formation of microthrombi, which may result in spontaneous hemorrhages within the brain and spinal cord. Although the precise mechanism remains unclear, it is believed that endothelial dysfunction and cytokine storms significantly contribute to these hemorrhagic complications, increasing the risk of severe neurological outcomes in individuals with COVID-19 [21]. A profound coagulation abnormality is caused by inflammation-induced changes in coagulation in combination with severe endothelial cell injury, with the consequent massive release of von Willebrand factor and plasminogen activators [22]. Coagulopathy is a possible symptom of endothelial cell inflammation and cytokine storms, which can lead to the production of microthrombi. Excessive and fast blood cytokine release, such as that of IL-6 and IL-1β, is indicative of a severe hyperimmune reaction [23]. When combined with viral endotheliopathy, IL-6 enhances vascular permeability and activates the coagulation system [22,23]. Vascular “neutrophilic plugs” comprising neutrophils and/or platelets, as well as neutrophil extracellular traps, are mostly triggered by IL-1β [23]. Vascular endothelial injury is a significant contributor to venous thrombus formation, particularly in the context of COVID-19. When the endothelium is damaged, it triggers a cascade of procoagulant events, including the activation of platelets and the release of tissue factors that promote clot formation. This process is further exacerbated by the inflammatory response associated with COVID-19, which increases the risk of thrombosis. In severe cases, this can lead to serious complications, including venous thromboembolism, which may manifest as deep vein thrombosis or pulmonary embolism [24].

Pituitary apoplexy is a clinical entity; the presence of hemorrhage in a sellar tumor is not the same as apoplexy. In our patient, a previously undocumented bitemporal hemianopia was present. Finding the underlying mechanisms is therefore extremely relevant to clinical practice. TNFα is an essential cytokine that mediates different hemorrhagic events by increasing vascular permeability, destroying parts of the vascular basement lamina, and upregulating VEGF and MMP-9. It is also involved in pituitary apoplexy [25,26]. Few cases of PA were reported in association with COVID-19 infection due to the expression of the ACE2 receptor [25]. Pituitary enrollment was considered an emergency in the COVID-19 time. Some hypotheses have been proposed to elucidate this clinical representation, including cross-reactivity between SARS-CoV-2 and pituitary proteins, COVID-19-associated coagulopathy, infection-driven acutely increased pituitary blood demand, as well as anti-platelet factor 4/heparin antibody progress after vaccination [26].

Hussain et al. presented a clinical case of pituitary apoplexy in a patient with a pituitary adenoma who also developed a COVID-19 infection [9]. Despite appropriate medical intervention, the patient succumbed to severe respiratory complications caused by COVID-19-associated pneumonia. This case report underscores the clinical challenges in managing pituitary apoplexy when compounded by severe respiratory complications from the viral infection, significantly worsening the prognosis and complicating the patient’s treatment [9]. Hazzi et al. investigated the relationship between COVID-19 infection and pituitary apoplexy through a case series and a literature review [10]. The authors suggest that COVID-19-induced coagulopathy may play a crucial role in the development of hemorrhage or infarction within the pituitary gland, leading to pituitary apoplexy, particularly in patients with pre-existing pituitary adenomas. The study explores the hypothesis that COVID-19 could act as a precipitating factor for pituitary apoplexy, emphasizing the importance of considering this virus as a potential trigger in patients with pituitary tumors [10].

Hussain et al. [9] and Hazzi et al. [10] primarily focused on pituitary apoplexy, but the case we report presents a more complex scenario, involving both an AVM and a pituitary macroadenoma in the context of COVID-19 infection. This patient exhibits a unique and more fatal combination of pathologies, where AVM-related hemorrhages and pituitary apoplexy are intertwined with COVID-19 infection.

## Conclusions

We present a rare case of a patient with a ruptured AVM and a pituitary macroadenoma exhibiting hemorrhage and infarct-like changes on pathology, alongside a COVID-19 infection with lung involvement and rapidly progressing complications. The convergence of these three conditions led to the patient’s rapid deterioration and subsequent death, despite the medical treatment provided. There are two vascular pathologies that express the same mechanisms of action; however, one due to vascular damage in the AVM was greater, facilitating hemorrhage with ventricular breakthrough, coagulopathy, inflammatory and secondary ischemic changes, as well as necrosis foci in pituitary adenoma.
